# Supplementary material for: A Preoptic Neurocircuit That Modulates Metabolic Flexibility
Source: bioRxiv. 2026 Feb 14:2026.01.15.699760. Preprint. [Version 2] doi: 10.64898/2026.01.15.699760 (PMC12918882; doi:10.64898/2026.01.15.699760)
Supplement: Supplement 1 [file NIHPP2026.01.15.699760v2-supplement-1.pdf]

bioRxiv preprint doi: https://doi.org/10.64898/2026.01.15.699760; this version posted February 14, 2026. The copyright holder for this preprint (which was not certified by peer review) is the author/funder, who has granted bioRxiv a license to display the preprint in perpetuity. It is made available under aCC-BY 4.0 International license.

Supplementary Figures

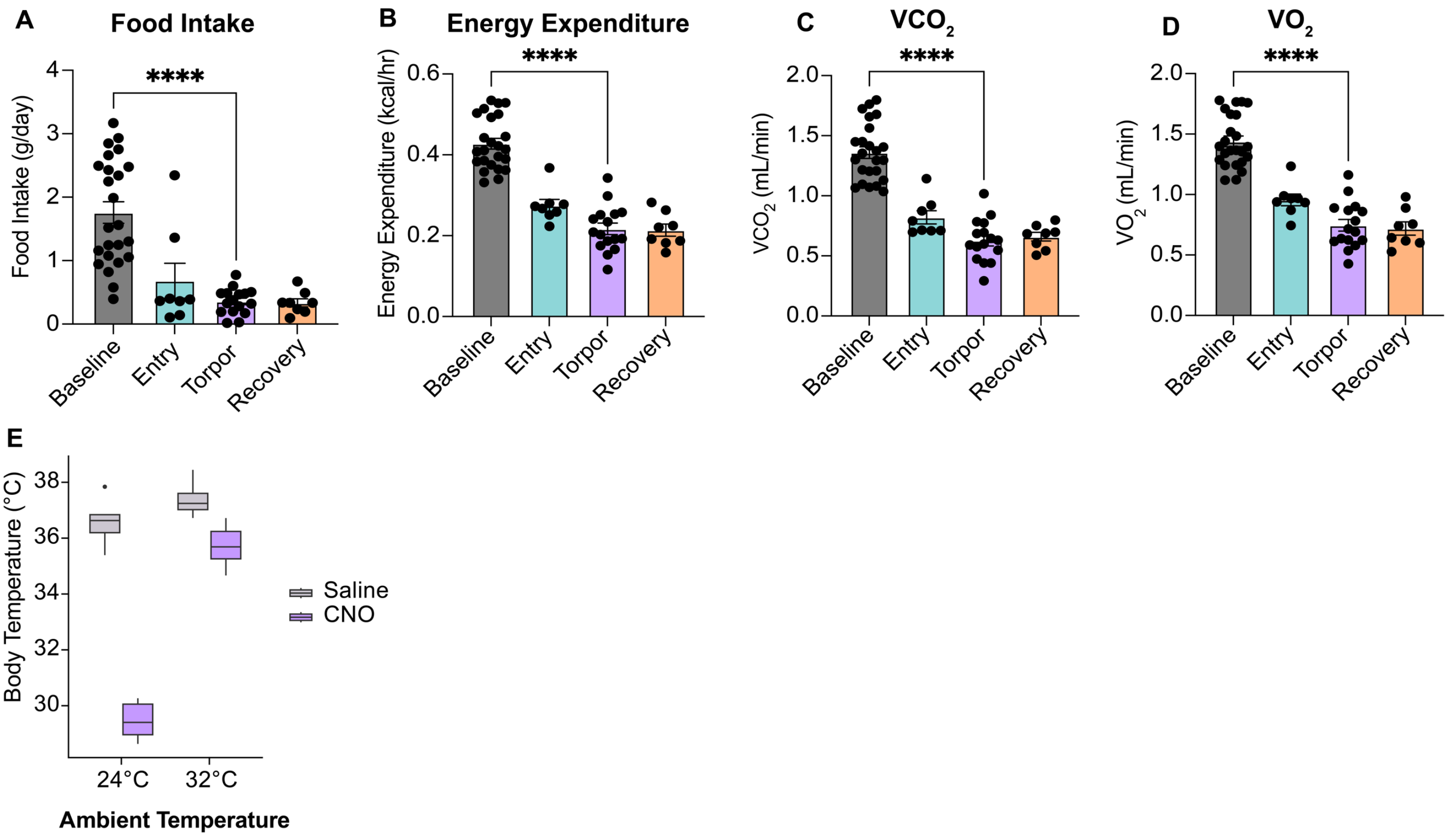

Supplementary Figure 1: avPOA stimulation regulates diverse metabolic parameters. (A-D) Quantitation of (**A**) Food Intake, **(B)** Energy Expenditure, **(C)** VCO_2, and (**D)** VO_2 throughout different phases of avPOA stimulation described in **Figures 1c-f**. Baseline = pre-CNO stimulation, Entry = 60 minutes post-CNO stimulation, Torpor = duration of torpor bout, Recovery = post-torpor period before returning to euthermia (**** = $p < 0.00005$, mean ±SEM, student's two-tailed t-test, n = 8 WT C57Bl/6J mice). **(E)** Quantitation of animal body temperature following 300 minutes of saline or Gq-DREADD stimulation at either T_a =24°C or 32°C (n = 8, mean ±SEM).

bioRxiv preprint doi: https://doi.org/10.64898/2026.01.15.699760; this version posted February 14, 2026. The copyright holder for this preprint (which was not certified by peer review) is the author/funder, who has granted bioRxiv a license to display the preprint in perpetuity. It is made available under aCC-BY 4.0 International license.

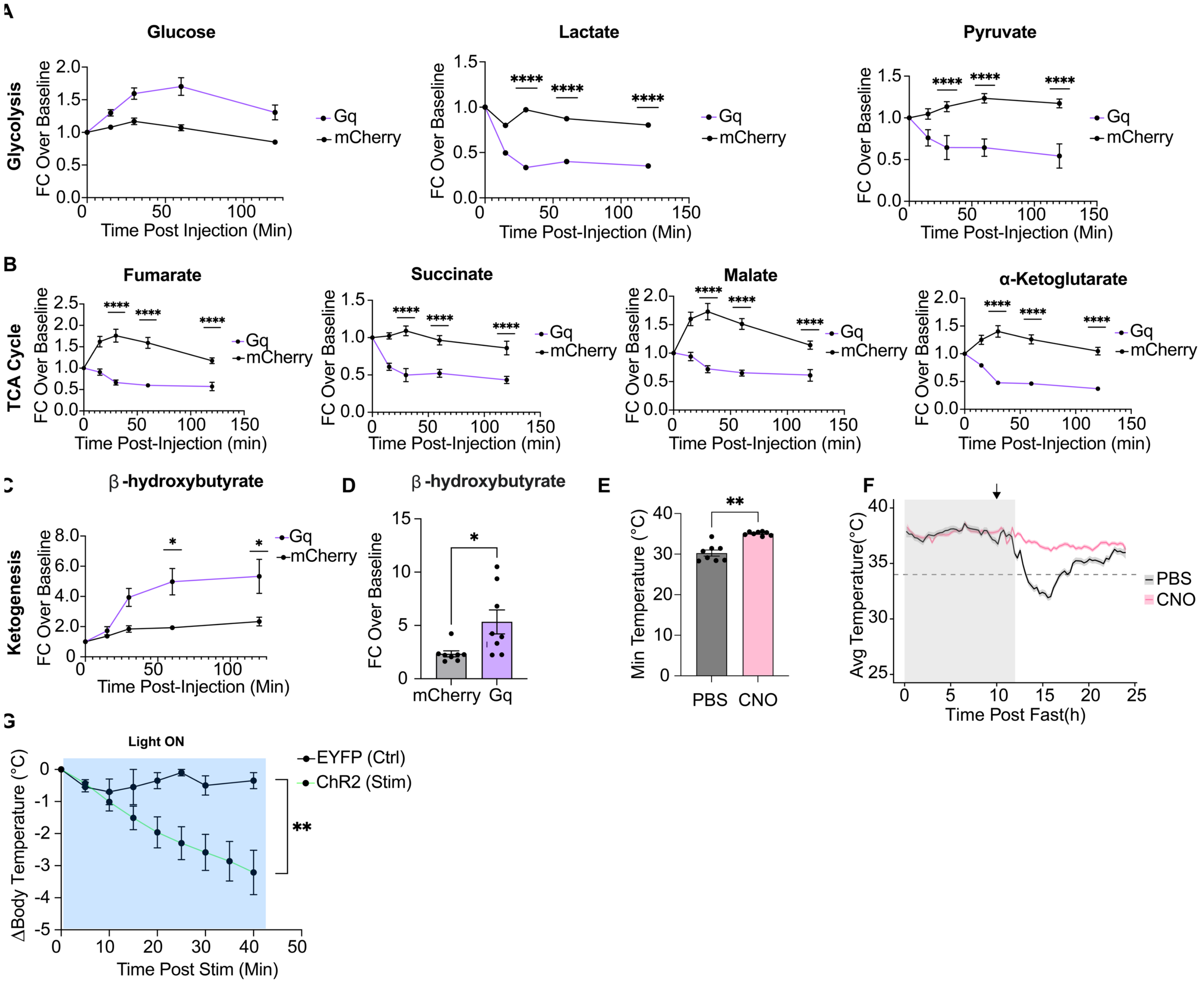

Supplementary Figure 2: TCA cycle and glycolytic metabolite abundance is altered following CNO stimulation. (A) Major glycolytic metabolites, including pyruvate and lactate, are monotonically decreased by CNO stimulation of Cre-dependent mCherry- or Gq-DREADD-transduced *Vglut2-IRES-Cre* mice as compared to their saline-injected counterparts. Conversely, glucose is monotonically increased upon CNO stimulation as compared to saline injection (** = $p < 0.005$, **** = $p < 0.00005$, student's two-tailed t-test, mean ±SEM, n = 8 animals). **(B)** Significantly downregulated TCA cycle metabolites include fumarate, succinate, malate, and α-ketoglutarate (**** = $p < 0.00005$, student's two-tailed t-test, mean ±SEM, n = 8 animals). Sample values reported as cohort-specific baseline-normalized fold change. Blood was collected

bioRxiv preprint doi: https://doi.org/10.64898/2026.01.15.699760; this version posted February 14, 2026. The copyright holder for this preprint (which was not certified by peer review) is the author/funder, who has granted bioRxiv a license to display the preprint in perpetuity. It is made available under aCC-BY 4.0 International license.

at 0, 15, 30, 60, 120 minutes post-injection. **(C)** β-hydroxybuyrate levels are significantly increased in CNO-injected animals as compared to controls (* = $p < 0.05$, student's two-tailed t-test, mean ±SEM, n = 8 animals). **(D)** Quantitation of (C) 120 minutes after CNO stimulation in mCherry and Gq animals (* = $p < 0.05$, student's two-tailed t-test, mean ±SEM, n = 8 animals). **(E)** Quantification of minimal T_b measured during fasting-induced torpor in Gi-DREADD-transduced *Vglut2-IRES-Cre* animals. Animals were fasted for 24 hours at the beginning of the dark cycle (ZT 12), and either PBS or CNO (10 mg/kg) was injected 10 hours after fasting began (ZT 22) (** = $p < 0.005$, student's two tailed t-test, n = 8 PBS, n = 8 CNO, same animals, mean ±SEM). **(F)** Longitudinal trace of animals in (E), demonstrating that Gi-DREADD animals injected with CNO do not go into torpor due to inhibition of $avPOA^{Vglut2}$ neurons, whereas PBS-injected animals enter normally. Arrow corresponds to time of CNO or PBS injection. Gray shading indicates dark cycle (ZT12-24). Horizontal dashed line at 34.5°C corresponds to the temperature threshold for torpor entry. **(G)** Stimulation of $avPOA^{Vglut2}$ neurons with 455 nm light decreases body temperature in ChR2-, but not EYFP-expressing animals over the 40-minute time period of blue light stimulation (* = $p < 0.05$, student's two-tailed t-test, mean ±SEM, n = 4 EYFP and n = 5 ChR2 animals).

bioRxiv preprint doi: https://doi.org/10.64898/2026.01.15.699760; this version posted February 14, 2026. The copyright holder for this preprint (which was not certified by peer review) is the author/funder, who has granted bioRxiv a license to display the preprint in perpetuity. It is made available under aCC-BY 4.0 International license.

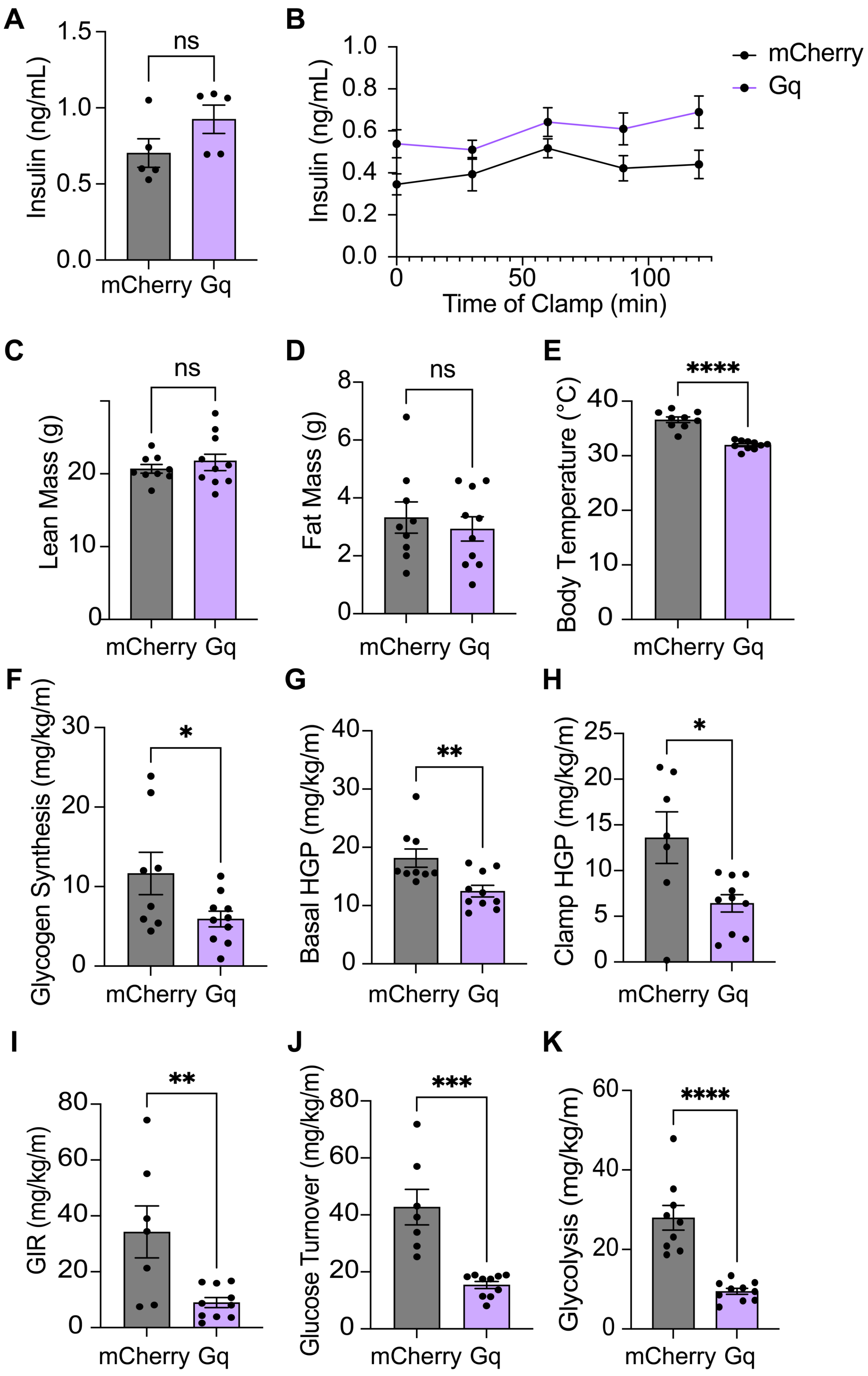

bioRxiv preprint doi: https://doi.org/10.64898/2026.01.15.699760; this version posted February 14, 2026. The copyright holder for this preprint (which was not certified by peer review) is the author/funder, who has granted bioRxiv a license to display the preprint in perpetuity. It is made available under aCC-BY 4.0 International license.

Supplementary Figure 3: Hyperinsulinemic-euglycemic clamp physical and clamp parameters. (A) Quantification of glucose and insulin levels in plasma of fed *Vglut2-IRES-Cre* animals expressing Cre-dependent mCherry or Gq-DREADD in the avPOA, 60 minutes post-CNO injection ($p<0.005$ = **). **(B)** Hyperglycemic clamp experiment monitoring plasma insulin levels for 120 minutes during administration of various concentrations of glucose. Blood samples were taken at 0, 30-, 60-, 90-, and 120-minute time points throughout the experiment (n = 9 mCherry, n = 10 Gq-DREADD animals). **(C-K)** correspond to the hyperinsulinemic-euglycemic clamp described in **Figure 3**. (**C**) Lean mass of animals analyzed by Dual X-ray Absorptiometry (DEXA) prior to beginning of the clamp. (**D**) Fat mass analyzed by DEXA prior to beginning of the clamp. **(E)** Average temperature of animals throughout the duration of the clamp. **(F)** Glycogen synthesis calculated during the clamp based on relative incorporation of ^{3}H into hepatic glycogen pool. (**G**) Hepatic Glucose Production (HGP) prior to initiation of the clamp as determined by the ratio of basal ^{3}H-Glucose infusion to the specific activity of plasma glucose prior to the initiation of the clamp. (**H)** Insulin-stimulated Hepatic Glucose Production during the clamp, determined by the difference between glucose infusion and whole-body glucose turnover rates. Insulin-stimulated HGP reflects the action of insulin on the liver specifically. **(I)** Glucose infusion rate (GIR) for each cohort. GIR was taken as an average over the duration of the experiment. Decreased GIR in Gq-DREADD-expressing animals indicates insulin insensitivity. Two control animals were excluded from analysis, as GIR measurements were highly irregular. **(J)** Glucose turnover in each cohort throughout the duration of the clamp, calculated as the ratio of ^{3}H-glucose infusion to plasma glucose specific activity during the final 30 minutes of the clamp. (**K)** Whole-body glycolysis estimated based on glycogen synthesis, GIR, and glucose clearance rates throughout the experiment (n = 9 mCherry, n = 10 Gq-DREADD, * = $p < 0.05$, ** = $p < 0.005$, *** = $p < 0.0005$, **** = $p < 0.00005$, two-tailed t-test).

bioRxiv preprint doi: https://doi.org/10.64898/2026.01.15.699760; this version posted February 14, 2026. The copyright holder for this preprint (which was not certified by peer review) is the author/funder, who has granted bioRxiv a license to display the preprint in perpetuity. It is made available under aCC-BY 4.0 International license.

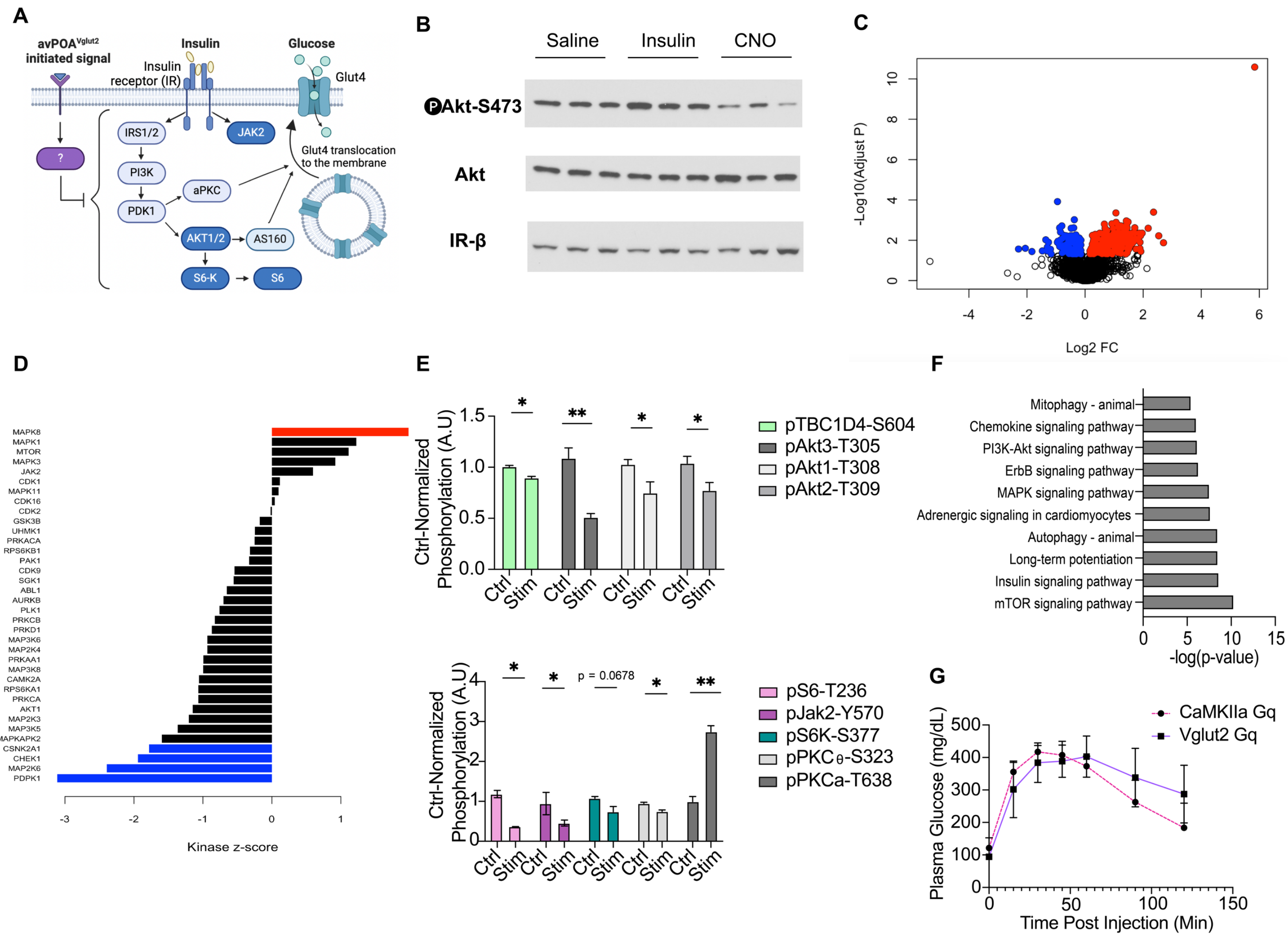

Supplementary Figure 4: Phosphoproteomic analysis of skeletal muscle reveals avPOA-induced defects in insulin signaling. (A) Schematic depicting the insulin signaling pathway and relevant downstream consequences of its activation with respect to glucose uptake. (**B)** Western blot of gastrocnemius muscle lysates obtained from mice injected with either saline, insulin, or CNO showing reduced Akt phosphorylation in CNO-injected animals as compared to saline-injected animals. **(C)** Volcano plot of differentially phosphorylated peptides in the gastrocnemius muscles of *Vglut2-IRES-Cre* mice transduced with Cre-dependent Gq-DREADD in the avPOA and injected with CNO as compared with saline. Significance threshold of $p < 0.05$ and Log_2FC of 0.6 used to determine significantly "hyperphosphorylated" or "hypophosphorylated" peptides. Red points correspond to peptides hyperphosphorylated in CNO relative to saline control, while blue correspond to hypophosphorylated peptides. **(D)** Kinase-substrate enrichment analysis score

bioRxiv preprint doi: https://doi.org/10.64898/2026.01.15.699760; this version posted February 14, 2026. The copyright holder for this preprint (which was not certified by peer review) is the author/funder, who has granted bioRxiv a license to display the preprint in perpetuity. It is made available under aCC-BY 4.0 International license.

of various kinases identified within the dataset in CNO vs. saline control animals. Kinases whose substrates demonstrated hyperphosphorylation in CNO vs. saline control muscles were scored higher, and those that were significant are shown in red. Kinases whose substrates demonstrated hypophosphorylation in CNO vs. saline control were scored lower, and those that were significant are shown in blue. Z-score cutoff of |1.5| was used to determine significance. **(E)** Saline-normalized phosphorylation of individual residues in key proteins in the insulin signaling pathway (n = 3 animals per cohort, * = $p < 0.05$, ** = $p < 0.005$, student's two-tailed t-test). **(F)** GO-Term analysis of peptides that demonstrated significant ($p < 0.05$) hypophosphorylation in CNO vs. control animals. **(G)** Comparison of the efficiency of avPOA^{Vglut2} stimulation during a GTT using a CaMKIIa-Gq as compared with a DIO-Gq (AAV-hSyn1-DIO-hM3D(Gq)-mCherry) used in B6 and *Vglut2-IRES-Cre* animals, respectively. No statistically significant difference was observed between the two cohorts.

bioRxiv preprint doi: https://doi.org/10.64898/2026.01.15.699760; this version posted February 14, 2026. The copyright holder for this preprint (which was not certified by peer review) is the author/funder, who has granted bioRxiv a license to display the preprint in perpetuity. It is made available under aCC-BY 4.0 International license.

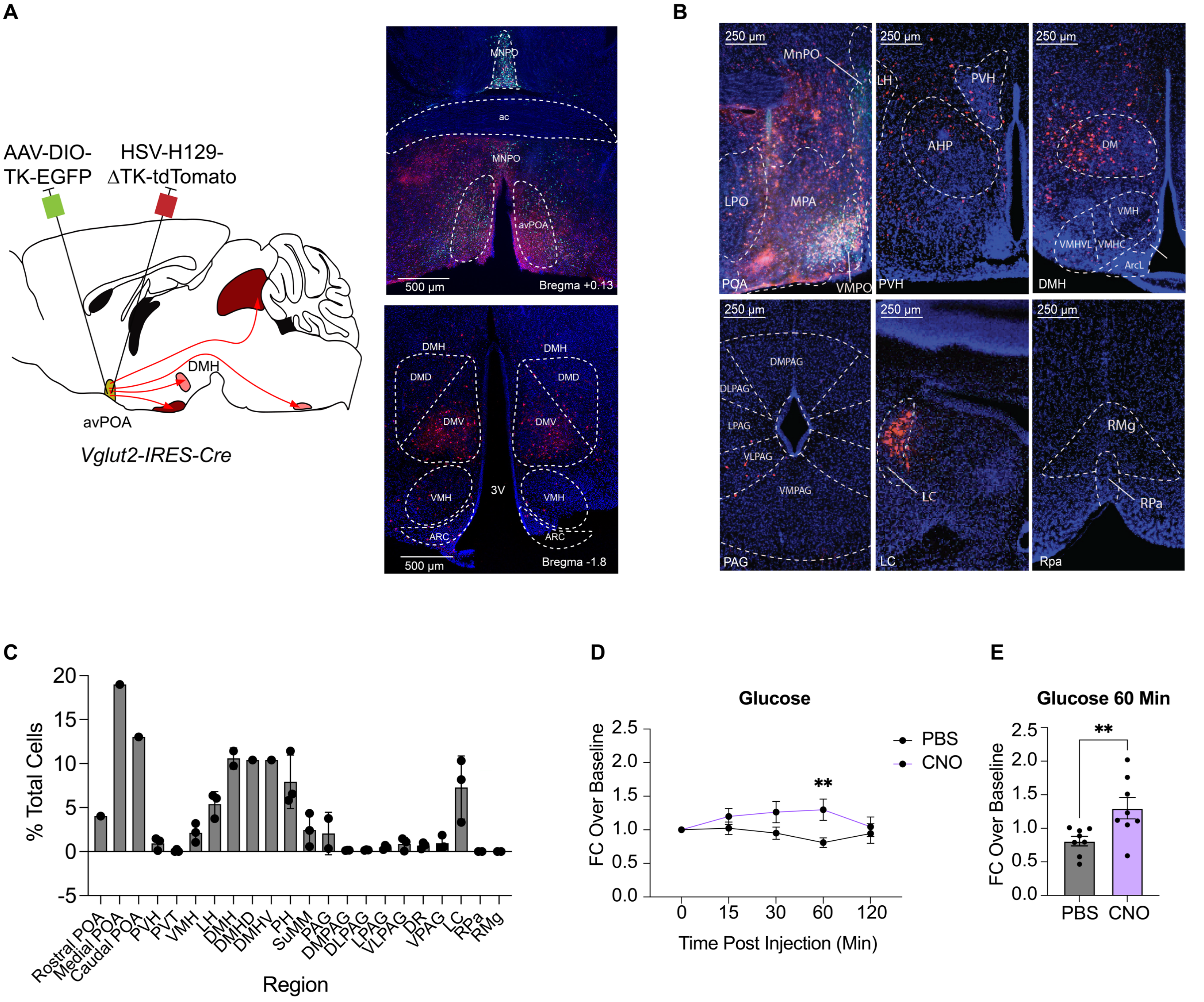

bioRxiv preprint doi: https://doi.org/10.64898/2026.01.15.699760; this version posted February 14, 2026. The copyright holder for this preprint (which was not certified by peer review) is the author/funder, who has granted bioRxiv a license to display the preprint in perpetuity. It is made available under aCC-BY 4.0 International license.

Supplementary Figure 5: avPOA neurons project to diverse regions, which may have distinct effects on body temperature and blood glucose. (**A**) (*Left*) Schematic for administration of AAV helper virus and HSV anterograde tracer to the avPOA of *Vglut2-IRES-Cre* animals. Mice were first transduced with a complementary helper AAV (AAV-DIO-TK-2A-EGFP), and after a two-week recovery period were transduced in the same region with HSV-H129-ΔTK-tdTomato, and sacrificed 65 hours following injection (n=3 *Vglut2-IRES-Cre* animals). (*Right*) (*Top*) Representative image of the starter region of the avPOA and (*Bottom*) one of the downstream targets of $avPOA^{Vglut2}$ neurons in the dorsomedial hypothalamus (DMH). HSV^{+} cells labeled in red, $AAV\text{-}DIO\text{-}TK\text{-}EGFP^{+}$ starter cells labeled in green. Note: in the DMH, HSV^{+} cells predominantly are observed in the ventral portion of the DMH (DMV). **(B)** Representative images of the same regions as **Figure 6a** in mice transduced with the anterograde monosynaptic tracer HSV-H129-ΔTK-tdTomato. (**C**) Quantitation of HSV expression in (B). Regions were defined in accordance with the Paxinos Reference Atlas, and the number of HSV-positive cells as a fraction of total DAPI cells was calculated for each region (n = 3 *Vglut2-IRES-Cre* animals, mean ±SEM). **(D)** Fold change in plasma glucose concentrations of *PACAP-2A-Cre* animals described in **Figure 6i** injected with either PBS or CNO at t = 0, 15, 30, 60, and 120 minutes post-injection relative to baseline (** = $p < 0.005$, student's two tailed t-test, n = 8 PBS, n = 8 CNO, mean ±SEM). **(E)** Quantification of glucose levels of individual animals from (D) at t = 60 minutes post-injection (** = $p < 0.005$, student's two tailed t-test, n = 8 PBS, n = 8 CNO, mean ±SEM).

bioRxiv preprint doi: https://doi.org/10.64898/2026.01.15.699760; this version posted February 14, 2026. The copyright holder for this preprint (which was not certified by peer review) is the author/funder, who has granted bioRxiv a license to display the preprint in perpetuity. It is made available under aCC-BY 4.0 International license.

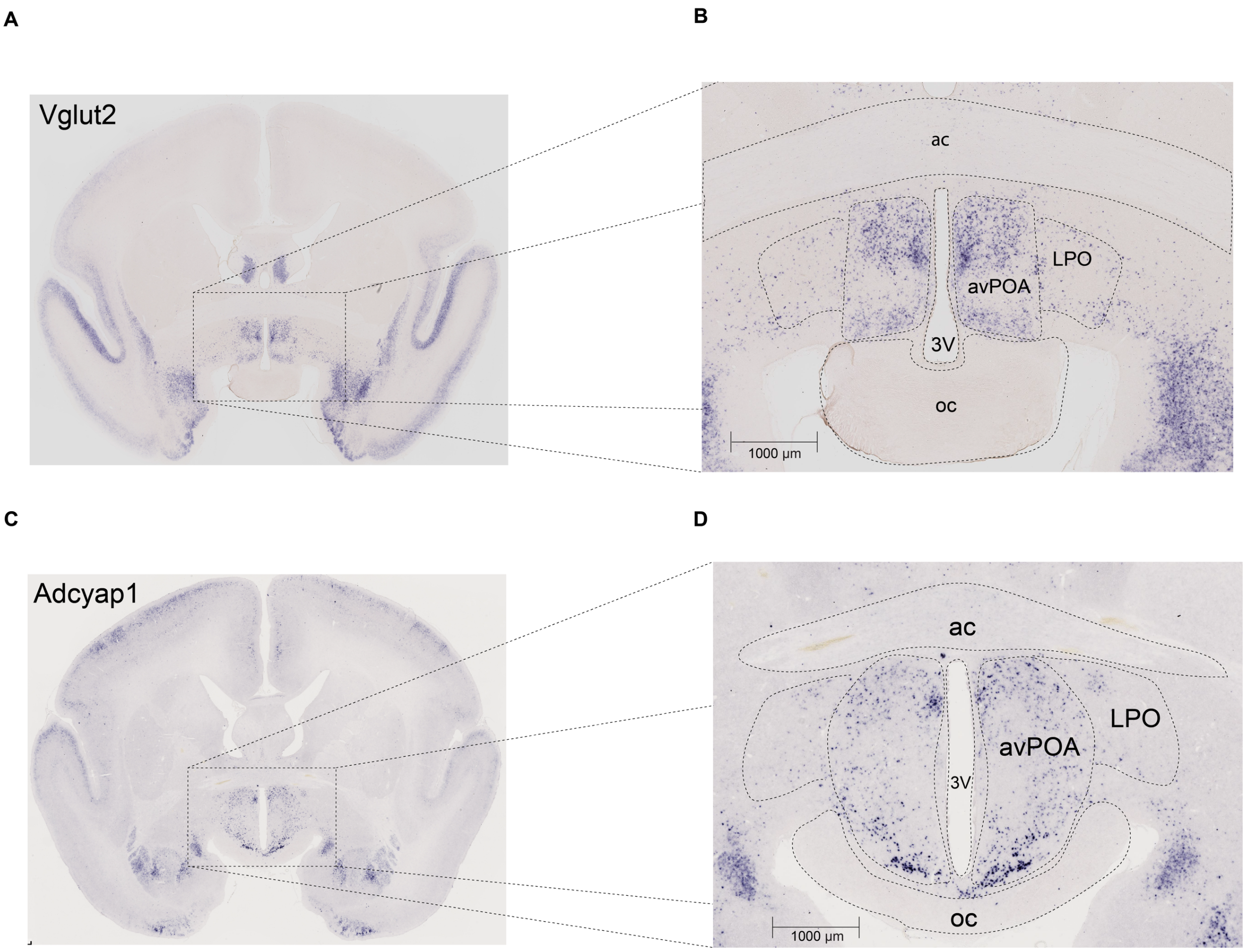

1290 **Supplementary Figure 6: avPOA expression of *Vglut2* and *Adcyap1* in the marmoset brain.**
1291 **(A)** RNA fluorescence *in situ* hybridization (FISH) image of *Vglut2* RNA expression in coronal
1292 marmoset brain region corresponding to mouse avPOA. Purple puncta indicate *Vglut2* RNA
1293 expression. **(B)** Magnification of (A) to show the avPOA and LPO regions that correspond to the
1294 mouse brain, demonstrating that avPOA^{Vglut2} expression is anatomically conserved between
1295 species. **(C)** RNA FISH image of *Adcyap1* RNA expression in coronal marmoset brain region
1296 corresponding to mouse avPOA. Purple puncta indicate *Adcyap1* RNA expression. **(D)**
1297 Magnification of (C) to show the avPOA and LPO regions that correspond to the mouse brain,
1298 demonstrating that avPOA^{Adcyap1} expression is anatomically conserved between species. Images
1299 adapted from: https://gene-atlas.brainminds.jp/
1300
